# Personal protective equipment and doffing procedures in out-of-hospital practice: assessment with a contamination simulation

**DOI:** 10.1186/s12245-021-00362-9

**Published:** 2021-07-13

**Authors:** Fabrice Pottier, Charles Groizard, Grégory Briche, Nicolas Haraczaj, Maxime Garnier, Vinciane Loones, Anna Ozguler, Michel Baer, Géraldine Baer, Thomas Loeb

**Affiliations:** 1 SAMU 92, APHP, 104 Boulevard Raymond Poincare, 92 380 Garches, France; 2 Division Criminalistique Physique et Chimie, Institut de Recherche Criminelle de la Gendarmerie Nationale, Cergy-Pontoise, France; 3 Département Environnement Incendie Explosifs, Institut de Recherche Criminelle de la Gendarmerie Nationale, Cergy-Pontoise, France; 4 Department of Emergency Medicine, Corporal Michael J. Crescenz VA Medical Center, University of Pennsylvania Medical Center, Philadelphia, PA USA

**Keywords:** Emergency medical service, Personal protective equipment, Simulation, Healthcare workers, Doffing

## Abstract

**Background:**

The use of personal protective equipment (PPE) by emergency medical services (EMS) providers requires specific attention, as it takes place in out-of-hospital unsecured settings. The aim of this study was to evaluate which PPE gown was less contaminating during doffing procedures in an EMS setting.

Six well-trained healthcare worker (HCW) subjects tested 4 different gowns: (1) surgical gowns (SG), (2) full body coveralls (FBC), (3) self-made alternative PPEs (SMP), and (4) non-surgical isolation gowns (NSIG). An invisible tracer was sprayed on the gown after donning each subject. After doffing, each HCW was photographed under UV lights to show areas of fluorescent “contamination” on their clothes. The number, size, and intensity level of contaminated areas were noted, as well as observational deviation from the procedure and doffing time. In addition, the subjects were asked to take a questionnaire about their perception of the level of comfort, ease of doffing, and overall safety for each gown.

**Results:**

Despite a well-trained team of HCW subjects, contamination while doffing was observed with every type of PPE gown, and with each HCW subject. All body areas were contaminated at least once, except the face. Contamination was more frequent while doffing FBCs. On the other hand, the removal of SG was found to be the least contaminating. The mean doffing time was significantly shorter with SG 1:29 and longer with FBC 2:26 (p=0.005).

**Conclusion:**

Results of this study converge towards the selection of surgical gowns over other types of PPE gowns, which met both contamination criteria as well as staff appreciation in this context. Specific attention should be paid to the legs and abdomino-pelvic areas. Additional protection such as protective trousers or aprons could be added.

## Background

Due to the highly contagious nature of SARS-CoV-2, and especially of its new variants, as well as its long-term persistence on inanimate surfaces, containment and prevention of spread are crucial in stopping the ongoing outbreak [1, 2].

One of the important aspects of maintaining an operational capacity of healthcare systems is preventing in-hospital transmission through the efficient use of personal protective equipment (PPE) by health care workers. PPE provides an essential barrier to limit the spread of the highly infectious agent, to prevent cross-contamination, and to protect healthcare workers (HCW) themselves [3].

The World Health Organization recommends that PPE for HCW include gowns, gloves, medical masks, and eye protection (goggles or face shields) [4].

In many countries, the pandemic context quickly led to a shortage of PPE. In acute care settings, this urged the selection of the most appropriate equipment among those available (in this specific situation), including “self-made” PPE, mostly for gowns and shields.

According to the specificity of each department, dedicated guidelines were developed. Wong et al. [5] described the modification of workflow and processes, the organization of PPE supply for staff, and the formulation of new clinical guidelines in operating rooms in a large hospital in Singapore. John et al. outlined specific protocols for PPE in an angiography suite [6]. Razzak et al. [7] proposed an estimation of US hospital workers’ infection rate according to different scenarios and concluded that PPE significantly reduces hospital workers’ infection. A recent Cochrane review stressed the need for simulation studies to determine which combinations of PPE and which doffing procedures result in the best protection [8].

At the emergency medical service (EMS) level, these procedures need particular attention as they take place in out-of-hospital unsecured settings, in emergency situations, with relatively shorter periods of time than in hospital settings. EMS professionals have to pay special consideration to the risk of contamination as there is no way to reorganize nor to reduce activity. They need to act rapidly and safely. A proper use of PPE under these circumstances is crucial; HCW must understand which PPE should be used and how to use it properly to be efficient within their setting with their own limited resources [9].

The aim of this study was to evaluate which PPE gown was the most protective and more specifically less contaminating during doffing procedure, in a simulated out-of-hospital context.

## Methods

### Population

Six well-trained EMS HCW (1 medical doctor, 1 certified nurse, and 4 ambulance drivers) volunteered to take part in the study. This study obtained MR-003 approval, as required by the National Council for Statistical Information (CNIL). Subjects gave their informed consent and were free to withdraw at any time. They were all part of the Exceptional Healthcare Situations (EHS) group created as a think tank to prepare and train HCW to mass casualties and unexpected events, including CBRN hazards. As part of this group, these HCWs actually design protocols, test, practice, and train other hospital workers in all aspects of PPE. This regular training program was set up in 2016, drawing from experience from the Ebola epidemic in West Africa, and is constantly updated according to scientific evidence and national/regional recommendations or laws from field authorities. In addition to organizing and practicing in regular monthly sessions, this group takes part in workshops organized by national experts in PPE procedures, and in large-scale simulation training exercises which takes place in France twice a year. All subjects in this study have been part of this group for 5 years. With regards for donning and doffing procedures were validated by this group of experts’ committee and formalized in our standards [10–16]. They comply with safety requirements for HCW protection. These procedures are based on WHO recommendations [4].

Typically, for any given EMS intervention, donning of HCW is performed in the ambulance upon arrival to the scene with the assistance of one paramedic. Doffing is performed at the very end of the medical intervention on hospital premises and without assistance.

HCW completed the simulation exercise, each testing all the different selected PPEs.

“Gendarmerie Nationale Research Institute” (IRCGN)—a forensic department of Gendarmerie Nationale—provided technical support with tracer, the location with specific rooms assisting staff, while EMS provided the PPE equipment, HCW subjects, study design, and investigators.

### The different gowns

Four different gowns were tested by each HCW subject (Table 1): (1) surgical gown (SG), (2) full body coverall (FBC), (3) self-made alternative PPE with plastic protection gear (SMP), and (4) non-surgical isolation gown (NSIG).

**Table 1:** Description of the four different gowns

	Surgical Gowns (1)	Full body coverall (2)	Self-made alternative (3)	Non Surgical isolation gowns (4)
Description	Surgical disposable scrub Surgical Gowns	Full Body coverall excepting face	Surgical disposable scrub Self-made PPE	Surgical disposable scrub Non surgical isolation Gowns
Opening & Doffing sides	Backside	Front side	Front side	Backside
Covered area	Gown with a scoop neck and covering down to lower legs	Full body including head, except face, hands and feet	Gown with a scoop neck and covering down to ankles	Neck to knee
Ties	- 1 bow at the waist - 1 scratch at the back of the neck	- 1 front zipper from neck to pelvic area - 1 adhesive tape to stick on top	- 1 pre-cut adhesive tape to stick on the front	- 1 belt with a back knot - 1 tie around the neck
Manufacturer	Bloc Opératoire Non Tissé GROUP’HYGIEN®	Tyvek® Dupont	Not Applicable	MEDICOM®
Reference	Commercial product meeting European standards EN13795	Commercial product compliant with ASTM F1670 & F1671 standards	Non-commercial made with polyane (60 micron polyethylene)	Commercial product conforming to ASTM D6978 standards
Picture				

Description of the 4 different gowns including opening and doffing sides, covered areas, securing mechanism, the manufacturer/commercial availability, and the descriptive picture. The rest of the personal protective equipment included 2 pairs of latex gloves, 1 Visor eyewear, 1 N95 facemask, 1 cotton sweatshirt and pants as underclothing, and personal shoes

The full equipment worn by each HCW subject in addition to the tested gowns consisted 2 pairs of latex gloves, 1 Visor eyewear, 1 N95 facemask, and 1 surgical hood. In addition, each wore similar clothes consisting of cotton sweatshirts and pants as underclothing and their own footwear (there was no buddy doffing).

### Protocol

The trials were performed indoors in a simulation room of the IRCGN. The order in which HCW subjects performed the procedure and with which gown was randomized (each volunteer had to draw lots in order to diminish bias).

**Fig. 1:**
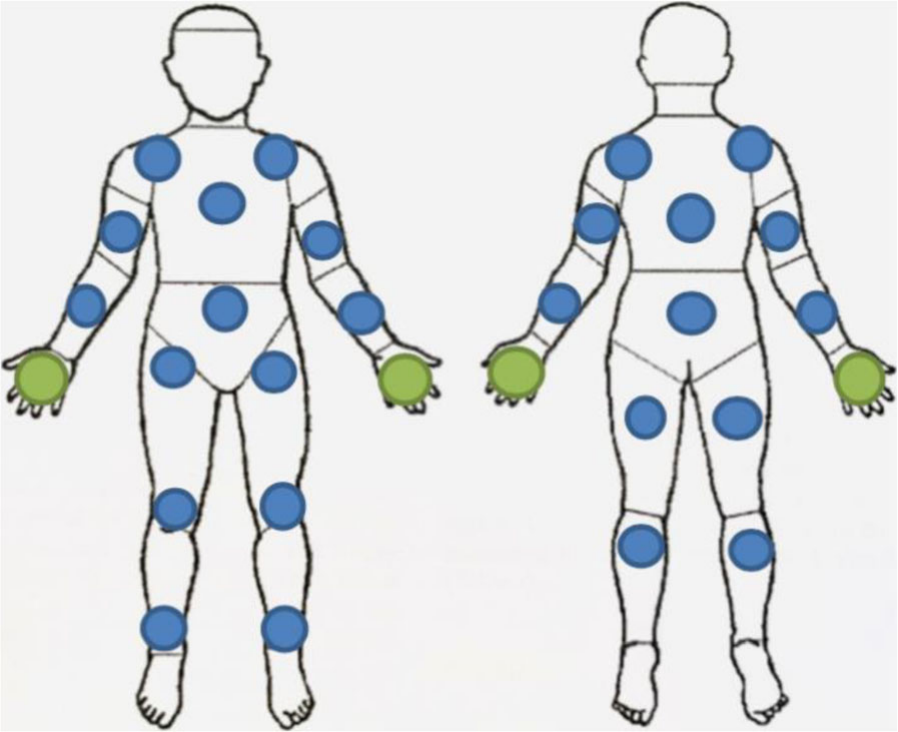
Body areas where the marking simulant was sprayed once (blue dots) or twice (green dots)

Donning was carried out in a space of 3 m^2^ (approximately 32 sq. ft.) which is approximately the ambulance space in which EMS personnel typically performs donning, and a buddy assisted each HCW. An invisible tracer with a very high potential for transfer, exclusively by contact, was then sprayed on the gown of the fully dressed HCW. Spraying was done in a specific room with a controlled environment, allowing reproducible target points, spraying distance, and lower variations due to ventilation. Figure 1 shows the different target points sprayed homogenously for each test on the various protected (ou gowned) body areas, once on specific sites on the whole body (blue dots) and twice on the hands (green dots).

The marker product was harmless, odorless, colorless, non-toxic, invisible to the naked eye, and revealed under UV light. IRCGN routinely uses this product for forensic activities in the form of a spray [17]. This marker closely simulated realistic contamination of PPE during doffing.

**Fig. 2:**
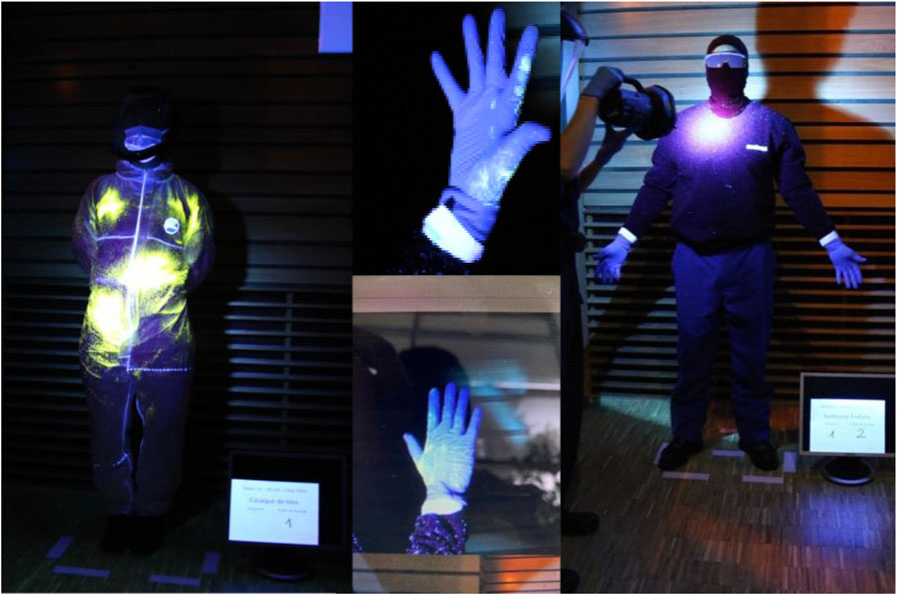
Use of UV light to reveal contamination on different body areas in a standardized way (at a downright angle, the sequential order of gown and passage order)

Two minutes after spraying, the HCW subject exited the room to an adjacent room and removed their PPE on their own. Screening was then performed using photographs under UV lights to show fluorescent “contamination.” The photographs were taken in a standardized way (Fig. 2)

After each sequence of donning and doffing, all equipment was disposed of in the usual manner, rooms were decontaminated, and HCW subjects changed their underclothing (gloves and post-test waste management).

Two investigator observers supervised the exercise and noted any deviation from the standard procedure for each of the 24 doffing tests; in addition, they measured the doffing time. Their observations were performed in consultation with each other.

Two independent experts also working hand in hand, blinded to the type of gown used, analyzed the pictures collected, marking the number, size, and intensity level of each contaminated area on a body map. There were 18 different body areas and 24 doffing procedures, making for a total of 432 potential contamination areas.

Finally, after each doffing procedure, each subject was asked to fill in a standardized survey containing questions about each gown. Questions consisted of visual analog scales ranging from 1 (totally disagree) to 10 (totally agree) regarding the level of comfort, ease of doffing, and level of protection perceived for each gown. In addition, they were asked to comment on whether they believed they had made any mistake or somehow self-contaminated themselves while doffing. All 24 questionnaires were filled out and collected.

### Data analysis

A total of 24 simulation tests were performed, as each of the 6 subjects tested each of the 4 gowns in a randomized order.

Contamination was evaluated by the experts, working hand in hand, according to 3 defined parameters:

- *Number* of contaminated body areas, with a maximum of 18 spots per test
- *Size* of contaminated area per body area (in cm^2^): each contaminated area was measured with the help of a graduated ruler and a body map
- *Intensity* level of contamination per body area, according to the stain intensity under UV lamp, reported as 1 = low, 2 = medium, and 3 = high intensity

Finally, doffing time and HCW subjects’ answers to post-simulation self-assessment regarding their opinion and perception of each gown were analyzed separately.

Comparisons were performed with non-parametric Kruskal-Wallis tests (SAS 9.4 Software).

## Results

### Number of contaminated areas

No test was free of contamination. In total, 76 contaminations occurred, out of 432 possible (17.6%) (Table 2). Legs and abdomino-pelvic areas were the most frequently contaminated body parts. Indeed, except in one HCW with one specific gown, legs were always contaminated regardless of the HCW subject or the type of gown. Abdomino-pelvic area was systematically contaminated with the FBC. On the other hand, the face was never contaminated regardless of the gown tested.

**Table 2:** Post doffing number of contaminated areas

	Face	Head	Shoulders	Upper arms	Forearms	Hands	Thorax	Upper back	Lower back	Abdomino-pelvic area	Thighs	Legs	Total
Surgical gown	0	1	1	0	0	2	0	2	1	1	2	7	17
Full body coverall	0	1	0	1	4	2	1	1	1	**6**	1	6	24
Self-made alternative PPE	0	0	2	5	1	2	0	0	0	2	1	5	18
Non-surgical isolation gown	0	2	0	0	0	3	2	0	0	1	2	7	17
Total	0/24	4/24	3/48	6/48	5/48	9/48	3/24	3/24	2/24	10/24	6/48	25/48	

Post doffing number of contaminated areas observed on all 6 volunteers according to the 4 different tested gowns

The divisor is the maximum number of potential contamination per body area

FBC was the most often contaminated type of gown with a total of 24 contaminated areas versus 17 for SG and NSIG, and 18 for SMP. Besides legs and abdomino-pelvic area, forearms were also often contaminated with FBC (4 times out of 12 potential contaminations).

Concerning all gowns except the FBC, besides upper arms (5 times out of 12 potential contaminations for SMP), no other body area was specifically contaminated.

### Size of contaminated areas

Hands, thighs, and legs were the body parts with the largest surfaces of contamination. When considering the different gowns, NSIG induced more contamination on legs, thorax, and thighs areas with a total of 722 cm^2^, followed by FBC with 678 cm^2^ more specifically on hands (300 cm^2^) and legs. SG was the attire that had the smallest contaminated total area (226 cm^2^) (Table 3).

**Table 3:** Post doffing contaminated surface in cm^2^

	Face	Head	Shoulders	Upper arms	Forearms	Hands	Thorax	Upper back	Lower back	Abdomino-pelvic area	Thighs	Legs	Total
Surgical gown	0	4	30	0	0	12	0	15	10	20	6	129	226
Full body coverall	0	5	0	10	23	**300**	5	10	30	75	60	160	678
Self-made alternative PPE	0	0	45	149	5	11	0	0	0	31	152	40	433
Non-surgical isolation gown	0	30	0	0	0	51	240	0	0	5	115	281	722
Total	0	39	75	159	28	374	245	25	40	131	333	610	

Post doffing contaminated area in cm^2^ observed on all 6 volunteers according to the 4 different tested gowns

### Intensity of contaminated areas

Abdomino-pelvic area and legs were the areas with the most intense stains on them. More specifically, FBC was intensively contaminated in these 2 areas, with a total of 15 out of 18 points for the abdomino-pelvic area (Table 4).

**Table 4:** Post doffing contamination intensity per body area

	Face	Head	Shoulders	Upper arms	Forearms	Hands	Thorax	Upper back	Lower back	Abdomino-pelvic area	Thighs	Legs	Mean intensity
Surgical gown	0	3	2	0	0	6	0	3	1	3	3	**19**	2.2
Full body coverall	0	3	0	3	11	6	2	3	3	**15**	6	18	3.9
Self-made alternative PPE	0	0	6	14	3	6	0	0	0	6	2	14	2.8
Non-surgical isolation gown	0	6	0	0	0	9	4	0	0	3	4	9	1.9
Total	**0/72**	**12/72**	**8/144**	**17/144**	**14/144**	**27/144**	**6/72**	**6/72**	**4/72**	**27/72**	**15/144**	**60/114**	

Post doffing contamination intensity per body area is observed on all 6 volunteers according to the 4 different tested gowns. The intensity level was parted in 1 = low, 2 = medium, and 3 = high intensity per body area. Mean intensity corresponds to the total of intensities per body area and per gown and is divided by 18 (total number of body areas). The divisor is the maximum number of potential contaminations per body area and intensity level

### Self-assessment

The 6 subjects found that all gowns were comfortable. Their ranking ranged from 8.8/10 for SG to 7.5 for SMP. Ease to remove was ranked from 9.0/10 for SG to 6.3 for SMP. The level of perceived protectiveness with the gowns ranged from 8.8/10 for SG and FBC and 7.2 for SMP and NSIG. Their self-perception of mistake and/or contamination while doffing was more frequent with FBC (4/6) and less frequent with SG (1/6) (Table 5).

**Table 5:** Answers of the 6 volunteers concerning their perception of different gowns and doffing time recorded

	Mistake while doffing (yes)	Contamination while doffing (yes)	Doffing time (mean time in min)	Comfort*	Ease to remove*	Safety*	Average score of comfort, ease to remove, and safety
Surgical gown	1/6	1/6	01:29	8.8	9.0	8.8	8.9
Full body coverall	4/6	4/6	02:26	8.7	6.5	8.8	8.0
Self-made alternative PPE	2/6	3/6	02:06	7.5	6.3	7.2	7.0
Non-surgical isolation gown	2/6	2/6	02:06	7.7	7.2	7.2	7.4
P			**0.005**	0.085	0.364	0.059	

*On a scale from 1 (totally disagree) to 10 (totally agree)

Answers of the 6 volunteers concerning their opinion on a scale from 1—totally disagree—to 10—totally agree—on each tested gowns with regard to mean comfort, ease to remove, and confidence self-estimated scores; average score of comfort, ease to remove and safety of their self-perceived opinion about potential mistake and contamination while doffing (yes or no); mean measured time for doffing

### Doffing time and comments from investigators

The mean doffing time was significantly shorter with SG (1:29) and longer with FBC (2:26) (p=0.005) (Table 5).

The investigator observers noted 4 times where subjects forgot to remove one or 2 gloves while doffing that took place once with full FBC and SG and that resulted in both hands contamination each time. This happened twice with NSIG, with just one hand contaminated once.

## Discussion

Despite a well-trained team of HCW subjects, contamination—specifically of the legs—upon doffing, was observed whatever the PPE gown or whoever the subject was. All body areas were at least once or more contaminated, except the face. Contamination was more frequent while doffing a FBC. On the other hand, the removal of SG remained the least contaminating.

The definition of contamination in itself varied here as 3 different aspects were considered: contamination on a dedicated body area, measure in cm^2^ of contaminated surface area, and intensity level per contaminated body area.

For these 3 parameters, the FBC had the worst or second worst scores. In addition, this attire was found to take the longest time for doffing.

In contrast, the SG was the most popular gown with the HCW subjects, had the lower “contamination” scores, and needed the shortest time for doffing.

Contaminated hands can easily be washed or decontaminated with hand sanitizer. The face was rarely contaminated, and this was encouraging because that meant that ingestion of virus while eating afterwards was unlikely, assuming that the hands were washed beforehand. However, contamination of other body parts was problematic because of the possibility of the spread of the virus to other surfaces and/or people.

This survey was performed in the most standardized possible way, in order to avoid bias due to environmental differences, sequence order, or contamination procedure variation. The spraying on many points of the body and the use of a marker with a very high transmissibility potential was meant to represent viral contamination which obviously would probably be less in real life with an actual viral or bacterial agent.

In addition, the training level of HCW was quite optimal and homogenous in order to avoid differences in PPE doffing techniques that could influence contamination levels. Kang et al. [18] noted that regardless of the different levels of PPE sets or various style combinations, frequent contaminations after PPE use and doffing occurred and were associated with poor HCW PPE techniques, knowledge deficits, and behavioral flaws. They emphasized on the need for refining PPE protocols, reinforcing PPE training, and improving and standardizing PPE equipment for targeting HCW optimal use.

The marker used for this survey has already been used by IRCGN for other purposes. Usage of UV light to reveal contamination was also used by Poller & al [19]. and Hall et al. [20] in a couple of articles where they evaluated PPE attires and doffing protocols in different scenarios. For training purposes, they used healthcare mannequins adapted to deliver simulated bodily fluids containing UV-fluorescent tracers. In their survey, they noted significant contamination from different exposure events.

Hall et al. [20] compared 6 PPE attires in different scenarios. They concluded the need to design a unified PPE attire and doffing procedure, incorporating the most protective PPE considered for each body area.

Another aspect to consider which may also play a role is familiarity with a specific gown. Baloh [21] explained that efforts to improve HCWs’ doffing performance needed to address HCWs’ preferences for both safety and expediency when using PPE, which has implications in PPE design, training approaches, and hospital policies and procedures.

Indeed, the use of a specific gown depends on each hospital’s supplies and policies. The specific pandemic context induced a significant shortage of PPE urging to find alternative solutions. There are few or no comparison of gowns in the literature, making it difficult to compare our results to those of others.

While airborne transmission of SARS-CoV-2 is the main mode of contamination, contact transmission via a contaminated surface was widely reported early in the pandemic. While it is important to consider all methods of transmission, a clinically significant risk of contact transmission from the surface was assumed on the basis of studies that have little resemblance to real-life scenarios and has probably been overestimated. With the benefit of hindsight, transmission by fomites (inanimate surfaces or objects) is actually not a major concern with the SARS-CoV-2. However, even if this risk is limited, periodically disinfecting surfaces, and properly using PPE, are still important and standard precautions [22]. Many pathogens other than SARS-CoV-2 can be transmitted by fomites. These include enteroviruses such as norovirus and rotavirus, human respiratory viruses as rhinovirus and adenovirus, or bacteria such as *Enterococcus faecium*, *Staphylococcus aureus*, or *Pseudomonas aeruginosa* [23].

## Conclusion

The constraints and technical difficulties met during doffing such as stripping from the front, from the back, or winding techniques might explain why some attires were more or less contaminant. This was in total accordance with subjects’ ratings and appreciations. Indeed, all results of this survey converge towards the selection of SG as the most protective PPE gown to be used by EMS workers. It met both contamination criteria as well as staff appreciation. The promising FBC showed some weak points in this survey, such as doffing time being too long.

The doffing procedure itself is an important step with high implications in the level of protection achieved by PPE. Specific attention should be paid to the legs and abdomino-pelvic areas. Additional protection such as protective trousers or apron could be added.

## Data Availability

All data generated or analyzed during this study are included in this published article

## Abbreviations

PPE: Personal protective equipment
EMS: Emergency medical service
HCW: Healthcare workers
SG: Surgical gown
FBC: Full body coverall
SMP: Self-made alternative PPE
NSIG: Non-surgical isolation gown
UV lights: Ultraviolet lights
CNIL: National Council for Statistical Information
EHS: Exceptional Healthcare Situations
CBRN: Chemical, biological, radiological, and nuclear
IRCGN: Gendarmerie Nationale Research Institute
FFP2: Filtering facepiece particles class 2
